# Real-world decision-making for post-exposure management of drug-resistant TB

**DOI:** 10.5588/ijtldopen.26.0125

**Published:** 2026-06-15

**Authors:** C.D. October, B. Beko, A. Reuter, V. Rennie, N. Ismail, E.C. Conceicao, S. Tonsing, R.M. Warren, S.T. Malherbe, A. Van Rie, L. Viljoen

**Affiliations:** 1 Division of Immunology, Faculty of Medicine and Health Sciences, Stellenbosch University, Cape Town, South Africa;; 2 Family Medicine and Population Health, Faculty of Medicine and Health Sciences, University of Antwerp, Antwerp, Belgium;; 3 DSI-NRF Centre of Excellence for Biomedical Tuberculosis Research, SAMRC Centre for Molecular and Cellular Biology, Division of Molecular Biology and Human Genetics, Faculty of Medicine and Health Sciences, Stellenbosch University, Cape Town, South Africa;; 4 Centre for Epidemic Response and Innovation (CERI), School for Data Science and Computational Thinking, Stellenbosch University, Cape Town, South Africa;; 5 Desmond Tutu TB Centre, Department of Paediatrics and Child Health, Faculty of Medicine and Health Sciences, Stellenbosch University, Cape Town, South Africa.

**Keywords:** tuberculosis, South Africa, TB preventive therapy, health care worker, qualitative research, Situated Clinical Decision-Making Framework

## Abstract

**BACKGROUND:**

Effective post-exposure management is essential to control drug-resistant TB (DR-TB). However, little is known about how health care workers (HCWs) make decisions on post-exposure management in resource-constrained settings.

**METHODS:**

We conducted a qualitative study using semi-structured interviews with 22 HCWs in the Western Cape, South Africa. Data were analysed thematically and interpreted using the Situated Clinical Decision-Making Framework (SCDMF) to explore how contextual conditions, knowledge, and clinical cues influence judgement and action in practice.

**RESULTS:**

Four interrelated themes were identified: crowded living conditions in informal settlements; stigma and non-disclosure of TB status; incomplete or delayed clinical information; and limited formal training on DR-TB post-exposure management. These factors constrained HCWs’ access to reliable social and clinical cues, limited their ability to assess exposure risk, and made consistent application of guidelines challenging. In response, HCWs relied on experiential knowledge, clinical intuition, and peer consultation to manage uncertainty. Real-world conditions frequently diverged from the SCDMF’s assumptions of enabling contexts, timely information, and accessible professional knowledge.

**CONCLUSION:**

HCWs’ decision-making for DR-TB post-exposure management is shaped by systemic constraints that necessitate adaptive but informal practices. Strengthening diagnostic turnaround times, formal training, and context-sensitive strategies to address stigma is critical to support consistent, evidence-based post-exposure DR-TB care.

Roughly 1.8 billion people are estimated to be infected with *Mycobacterium tuberculosis* and approximately 10.7 million people develop TB disease each year.^1^ TB control strategies encompass both treatment and prevention, each of which is adversely affected by the occurrence of drug-resistant TB (DR-TB), including rifampicin-resistant TB (RR-TB).^2,3^ Compared to drug-sensitive TB (DS-TB), DR-TB treatment is more complex to diagnose and more costly to treat, which contributes to poor treatment outcomes, impacting patients, their household, and their communities.^4^ Post-exposure management is one of the pillars of prevention and includes the assessment and treatment of individuals who have been exposed to TB and administration of TB preventive therapy (TPT) when there is no evidence of active disease.^5^ Effective TPT requires knowledge of the index case’s resistance profile to select the optimal TPT regimen.^6^ In 2024, the WHO recommended 6 or 9 months of daily isoniazid, 3 months of weekly rifapentine plus isoniazid, or 3 months of daily isoniazid plus rifampicin for contacts of patients with drug-susceptible TB.^2^ For contacts exposed to multidrug- or rifampicin-resistant TB (MDR/RR-TB), 6 months of daily levofloxacin is recommended for TPT.

In 2023, South Africa changed to a ‘test and treat’ approach for DS-TB. While nominally the National Guidelines^7^ suggest a similar strategy for contacts of people with DR-TB, unlike for DS-TB, they lack the operational detail necessary for frontline clinicians to implement it effectively. Instead, providers are directed to the 2019 RR-TB guidelines which lack clarity on which people exposed to DR-TB should be offered TPT and suggest outdated multidrug TPT regimens that no longer align with best practices for DR-TB post-exposure care.

Health care workers (HCWs) face various challenges in post-exposure management, including low referral and TPT uptake, limited knowledge, and operational constraints.^8,9^ Because of this level of uncertainty, HCWs often draw on experiential knowledge and professional judgement.^10^ The Situated Clinical Decision-Making Framework (SCDMF) provides a lens to understand how contextual, cognitive, and experiential factors shape decision-making in complex clinical environments.^11^ It comprises context (micro, meso, macro), foundational knowledge, and critical thinking. In this study, we explored HCWs’ real-world decision-making using semi-structured interviews and interpreted findings through the SCDMF.

## METHODS

We conducted a qualitative study in 11 public health facilities in the Western Cape province of South Africa, a high TB and DR-TB burden setting. Nationally, there are approximately 658 DR-TB treatment sites^12^ and in 2022, ∼13,000 individuals were initiated on MDR/RR-TB treatment.^13^ As of 3 March 2026, 2,144 active DR-TB cases were recorded in the province.^14^ These facilities serve communities with poor socio-economic living conditions, high population density, and overcrowding^15^ where HCWs navigate complex clinical and social constraints. We selected medical officers, nurses, and community workers to present a range of genders, roles in TB care, and experience. A semi-structured interview guide was developed to collect information on experiences and challenges of current methods for post-exposure management for DR-TB. Interviews lasting about 45 min were conducted in English, Afrikaans, or Xhosa in private rooms at the public health facilities. Interviews conducted in English were transcribed verbatim using Trint’s transcription software.^16^ Interviews conducted in Afrikaans and Xhosa were translated and transcribed by researchers. HCWs were invited to participate until data saturation was reached, using a previously described method.^17^

### Data analysis

Data were analysed using Braun and Clarke’s six-step thematic analysis, integrating deductive and inductive approaches.^18^ We used the SCDMF theoretical framework to deduce themes and map responses to the context, foundational knowledge, and critical thinking processes. The analysis proceeded iteratively to allow for hypothesis-generating insights into HCWs decision-making processes. We complemented this with an inductive analytical step to capture emerging themes outside the SCDMF, using ATLAS.ti Version 25.0.1 (32922) to facilitate coding and to generate themes from the transcriptions.^19^

### Ethical statement

The study was approved by the Human Research Ethics Committee of the University of Stellenbosch (reference number: N24/01/002), City of Cape Town (project ID number: 12071), and the Western Cape Government Department of Health (WC_202407_003). All participants gave written informed consent.

## RESULTS

The 22 participants were mostly female (n = 20), had an average of 11 years of experience in DR-TB care, and included nine medical officers, nine nurses, and four community workers. Four main themes were identified: crowded living conditions in informal settlements, stigma and non-disclosure, incomplete clinical information, and lack of formal training for HCWs. Within these themes, components of the SCDMF were identified to explore HCWs’ decision-making for post-exposure management. The Figure shows how contextual influences at the micro-, meso-, and macro-levels shape the knowledge HCWs draw on, the clinical cues they attend to, and the judgement, action, and reappraisal processes through which post-exposure management decisions are made.

**Figure.**
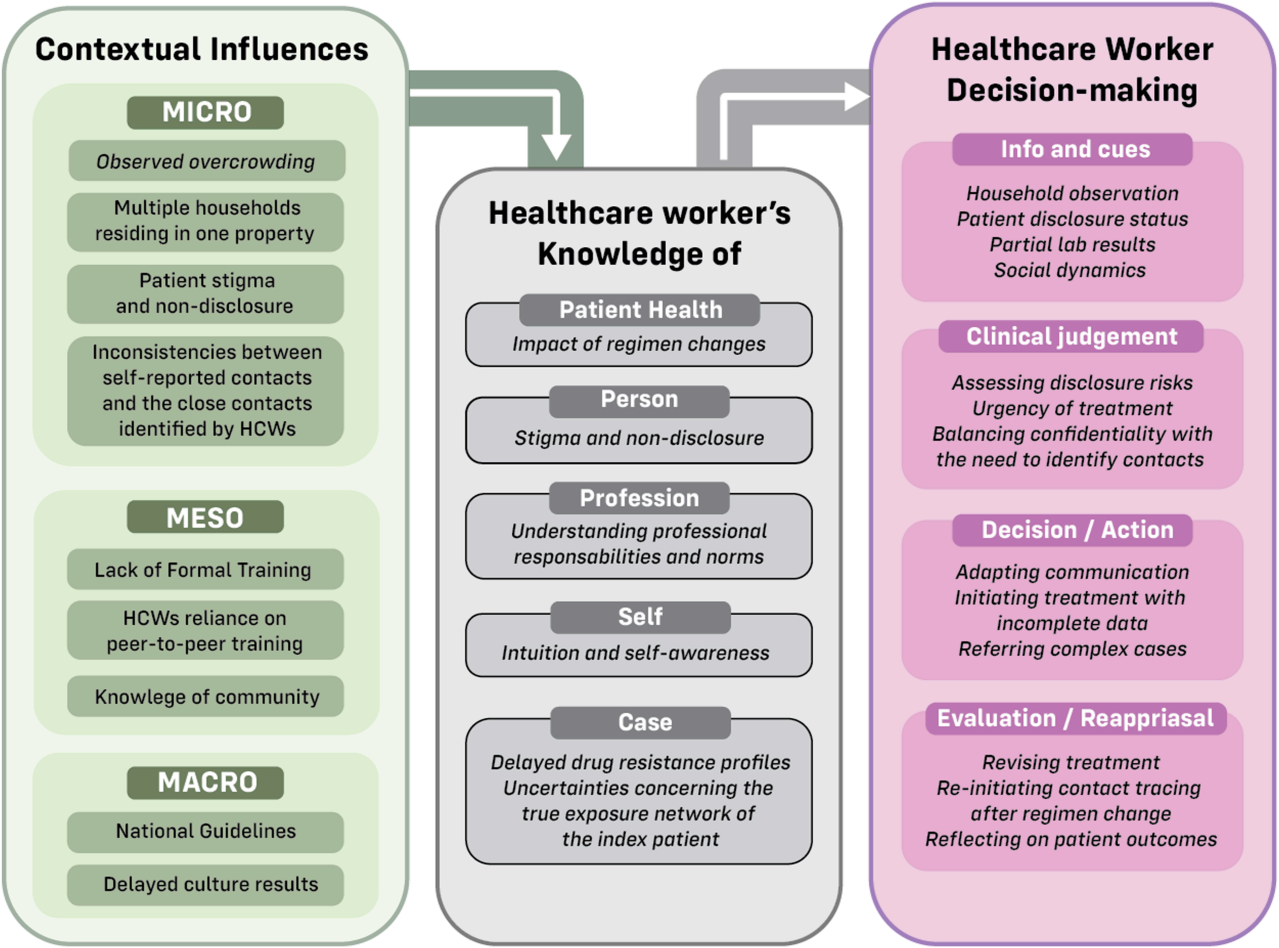
How contextual influences at the micro-, meso-, and macro-levels shape the knowledge and decision-making of health care workers (HCWs).

### Crowded living conditions in informal settlements

HCWs experience that patients living in informal settlements are often reluctant to disclose their DR-TB diagnosis to their contacts. Household and community members’ *context* (*micro*) of overcrowded housing and multiple households within a single property also results in inconsistencies between close contacts reported by patients and observations on likely contacts made during home visits.

In [this community] we’ve got a challenge of overpopulation... It’s about five houses in the same house, in the same yard, but it’s different families.

TB Nurse 3

### Stigma and non-disclosure

Patients often withhold information about contacts in the household, work, or community due to stigma, privacy concerns, fear of loss of housing, fear of rejection by family or colleagues, and misunderstanding of the purpose of tracing. This results in the underreporting of close contacts and thus HCWs’ poor knowledge of the case (contextual: micro, knowledge of the case).

Patients are afraid of what the family might think… They are staying in a shack on someone else’s property, so they are afraid those people are going to push them away.

TB Nurse 9

Since most of the people know that if you have TB, you might have HIV. You know, people are talking, especially in our townships.

TB Nurse 1

Work is the problem of confidentiality. … And to tell others about you… it depends how is the boss… If the boss has some tones that are harsh and attitudes, I’m [patient with TB] not sharing my disease.

TB Nurse 1

In these settings, HCWs’ decisions on who to identify as a contact are influenced by their knowledge of the community (contextual: meso). An example of this is when community workers conduct home visits for an index patient who has not yet disclosed their TB status (knowledge of the person), they rely on knowledge of the case to identify undisclosed household contacts.

In this context, HCWs become highly aware of their tone and presence (knowledge of the self) and the need to uphold confidentiality to avoid revealing sensitive information (knowledge of the profession). They navigate carefully and ethically and ask targeted questions to avoid jeopardising patient safety and relationships (judgement). They assess the household dynamic (cue) to decide what information can safely be shared (judgement) and to adapt their communication and behaviour to avoid unintentional disclosure (decision):

When you go do a home visit to do contact tracing, you have to be so careful what to say, when to say it, how to say it.

Community Worker 2

I first talk to the index… Do they know your status? If they don’t know, then I can’t just go.

Community Worker 1

### Slow and incomplete clinical information

When performing post-exposure management of DR-TB, HCWs often need to make decisions based on incomplete information (contextual: macro). Often, HCWs initiate TB treatment (judgement) and contact tracing based on partial laboratory results (cue). In this context, HCWs need to initiate RR-TB treatment in the index patient based on available cues and rely on experience-based intuition (knowledge of the self) to prioritise timely intervention over procedural precision. Uncertainty regarding the drug susceptibility profile of the index patient could result in delays in initiating appropriate TPT for their contacts (decision). They then adjust regimens once definitive results on drug resistance become available, which affects the timing and accuracy of decisions and the treatment outcomes in patients and their contacts (evaluation of outcomes). This type of decision-making affects patient care and contact tracing (evaluation of outcomes; knowledge of the patient), results in HCW frustration about providing ineffective treatment and having to change regimens, and is emotionally challenging for patients as they become tired of changing regimens and the prolonged period of taking medication (evaluation of outcomes).

Culture results [that allows phenotypic drug resistance testing] take approximately six weeks… doctors often start patients on TB treatment based only on smear results.

Medical Officer 2

I have two patients whereby we had rif unsuccessful … like it took us a month. Or more than six weeks for us to get the culture results. The culture results came back rif-resistant, and then now we must start over again.

TB Nurse 4

Sometimes you would start a patient and then the second line [drug susceptibility tests] come out, all results will come out and then you have to change it again.

Medical Officer 6

For the patient it’s difficult to understand that they need to change the medication… and then finding them again becomes an issue and then contact tracing changes again.

Medical Officer 2

### Lack of training

Post-exposure management for DR-TB is complex and evolving, making up-to-date knowledge, clear implementation guidance, policy guidance, and training essential for optimal patient outcomes. Only one of the 22 HCWs expressed that they had been trained on how to implement the DR-TB post-exposure algorithm outlined in the 2019 guidelines. Most (21/22) had not received any formal training on post-exposure management.

In this context (meso), decision-making shifts from formal to informal learning, peer consultation, and trusting one’s own resourcefulness. HCWs described periodic meetings and interactions with colleagues as opportunities to reinforce professional competence. They draw on professional norms, mentorship, and peer collaboration to maintain standards despite institutional training gaps (contextual: meso, knowledge of the profession). This demonstrates awareness of professional boundaries (knowledge of the profession), personal limitations (knowledge of the self), and clinical complexity (knowledge of the case).

We never had any trainings.

Community Worker 2

We learn as we go about it.

TB Nurse 1

With the MDR contacts, especially with children, we usually discuss it first with our supervisor and then if it’s a complicated case maybe there’s like XDR-TB then we will usually refer to Prof and he will manage from there and work out a regimen for us to start.

Medical Officer 4

## DISCUSSION

Our findings indicate that HCWs experience constraints, which requires them to rely on experiential knowledge, peer consultation, and judgement. These constraints are compounded by non-disclosure, arising from patients’ living circumstances and perceptions of stigma. Due to these issues, HCWs use adaptive decision-making and critical thinking to ensure that work remains feasible and ethical rather than ‘simply’ implementing guidelines. This is similar to reports from Ethiopia, where HCWs perceived DR-TB as an escalating public health threat resulting from health-system weaknesses alongside adverse socio-economic conditions, including poverty, poor housing, and overcrowding.^20^

The realities of DR-TB post-exposure management do not align with the SCDMF’s assumptions that HCWs have access to enabling context, robust foundational knowledge and timely cues, opportunities to develop knowledge of the patient and profession, and the ability to evaluate outcomes. In the real world, non-disclosure and stigma limits HCWs’ ability to develop knowledge of the patient’s household composition, close contacts, treatment history, and exposure risk. TB-related stigma is widely documented^21,22^ and contributes to patients’ reluctance to disclose their TB status due to associations with HIV and perceptions of uncleanliness and poverty.^23^ This non-disclosure obscures key social and exposure-related cues within the SCDMF, such as information on household members, which increases uncertainty in HCWs’ risk assessments and post-exposure management decisions. Stigma not only affects patients but also shapes HCWs’ clinical decision-making, encouraging them to adapt their behaviour to avoid inadvertent disclosure. Stigma actively shapes HCWs’ communication practices and ethical decision-making in community-based care in South Africa.^24^

When drug-susceptibility test results are delayed, HCWs may need to initiate treatment in the index patient, and provide post-exposure management to contacts prior to knowledge of index’s full drug-resistance profile.^25,26^ In such circumstances, HCWs compensate for gaps in foundational clinical knowledge by engaging in critical thinking and drawing on experience-based intuition to make reasoned judgements under uncertainty. This aligns with the literature on clinical decision-making under uncertainty, where experienced clinicians adopt strategies to overcome lack of optimal information^27^ and thus reflects rational adaptation. Such adaptations can have consequences as HCWs expressed that patients become confused and emotionally distressed when doctors change their treatment due to new drug-susceptibility test results.^28,29^ Gaps in training on post-exposure DR-TB management prevented HCWs to acquire knowledge. HCW experience of lacking training may also have been influenced by unclear policy guidelines on post-exposure management and TPT recommendations for DR-TB. HCWs relied on experiential learning and peer consultation, increasing dependence on individual judgement. This compensation mirrors findings from studies conducted during the COVID-19 pandemic, where interprofessional collaboration played a critical role in developing HCWs’ competence in rapidly changing and uncertain clinical environments.^30^ These adaptations are consistent with Benner’s application of the Dreyfus model,^31^ which suggests that progression toward proficiency is marked by an enhanced ability to interpret situational nuances when formal rules are insufficient. Gaps in formal training contributes to practice variation,^32,33^ reinforcing that experiential learning alone cannot substitute structured professional development. Improving DR-TB care requires context-adapted decision support tools, clearer post-exposure management guidelines, and strengthened training to enhance consistency and support HCWs’ decision-making under uncertainty.

Key strengths of this study are the inclusion of different cadres of HCWs and levels of the health system and the application of the SCDMF as an analytic framework for an in-depth exploration of how decision-making unfolds under real-world constraints. While these promote internal validity in the study setting, the findings may have limited external validity as they may not capture decision-making dynamics in other regions. As the analysis is based on self-reported accounts, findings may not fully represent observed clinical practice. Findings are limited to HCWs’ reported experiences. However, the in-depth, iterative approach supports credibility.^34^ Future research incorporating patient perspectives or programmatic data could enable triangulation and strengthen robustness.^35^ Furthermore, the study did not systematically examine access to or use of written guidelines or policy documents, which may also influence decision-making. Despite these limitations, the findings provide valuable insight into how systemic constraints shape clinical decision-making in post-exposure management for DR-TB.

## CONCLUSION

This study provides novel insight into how contextual and systemic challenges shape HCWs’ decision-making in post-exposure management for DR-TB, revealing the adaptive strategies they employ when standard guidelines and formal training are insufficient. Improving DR-TB post-exposure management therefore requires simultaneous interventions that restore the conditions necessary for effective HCW decision-making. Without such system-level interventions, post-exposure management of DR-TB will continue to depend on informal solutions, risking inconsistency, patient distress, and suboptimal prevention of DR-TB.
